# Acoustic Detail But Not Predictability of Task-Irrelevant Speech Disrupts Working Memory

**DOI:** 10.3389/fnhum.2016.00538

**Published:** 2016-10-25

**Authors:** Malte Wöstmann, Jonas Obleser

**Affiliations:** Department of Psychology, University of LübeckLübeck, Germany

**Keywords:** attention, memory, task-irrelevant speech, acoustic detail, predictability, age

## Abstract

Attended speech is comprehended better not only if more acoustic detail is available, but also if it is semantically highly predictable. But can more acoustic detail or higher predictability turn into disadvantages and distract a listener if the speech signal is to be ignored? Also, does the degree of distraction increase for older listeners who typically show a decline in attentional control ability? Adopting the irrelevant-speech paradigm, we tested whether younger (age 23–33 years) and older (60–78 years) listeners’ working memory for the serial order of spoken digits would be disrupted by the presentation of task-irrelevant speech varying in its acoustic detail (using noise-vocoding) and its semantic predictability (of sentence endings). More acoustic detail, but not higher predictability, of task-irrelevant speech aggravated memory interference. This pattern of results did not differ between younger and older listeners, despite generally lower performance in older listeners. Our findings suggest that the focus of attention determines how acoustics and predictability affect the processing of speech: first, as more acoustic detail is known to enhance speech comprehension and memory for speech, we here demonstrate that more acoustic detail of ignored speech enhances the degree of distraction. Second, while higher predictability of attended speech is known to also enhance speech comprehension under acoustically adverse conditions, higher predictability of ignored speech is unable to exert any distracting effect upon working memory performance in younger or older listeners. These findings suggest that features that make attended speech easier to comprehend do not necessarily enhance distraction by ignored speech.

## Introduction

Selective attention enables the cognitive system to select and prioritize relevant information from the environment and to filter out irrelevant information (Serences and Kastner, 2014). In the auditory modality, the focus of selective attention determines which sounds are ‘attended,’ while the remaining (unattended) sound items are considered ‘ignored’ (for a review article on auditory attention, see Fritz et al., 2007). Human speech is a particularly challenging sensory signal for selective attention, since it commonly occurs in the presence of competing talkers and environmental noise (Shinn-Cunningham, 2008; McDermott, 2009). When a speech signal is attended, more acoustic detail and semantic predictability both enhance speech comprehension (e.g., Obleser et al., 2007). But do these features similarly, increase the degree of distraction from a speech signal that is ignored? And, more importantly, do acoustic detail and predictability of to-be-ignored speech impede the processing of task-relevant speech in memory?

Manipulations of acoustic detail and semantic predictability have been widely used in neuroscientific studies on speech comprehension. In order to vary the acoustic detail of a speech signal, the *noise-vocoding* technique is frequently used, which parametrically degrades the spectral content (i.e., the fine structure) but leaves the coarse temporal structure (i.e., the temporal envelope) of the speech signal largely intact (for an overview of the signal processing involved in noise-vocoding, see Rosen et al., 1999). Critically, the use of fewer frequency bands used for noise-vocoding decreases speech comprehension (Shannon et al., 1995). High semantic predictability of sentence endings can have a ‘compensatory’ function under acoustically adverse conditions as it aids speech comprehension (Pichora-Fuller et al., 1995). When studied in combination, it has been shown that high predictability of sentence endings is most beneficial for speech comprehension under intermediate levels of noise-vocoding (Obleser et al., 2007; Hartwigsen et al., 2015). Importantly, the respective detrimental and beneficial effects of noise-vocoding and predictability have mostly been studied for speech signals that listeners were supposed to attend to. Here, we tested the hypothesis that those features that make attended speech easier to comprehend render ignored speech more distracting. We thus predicted that more acoustic detail but also higher predictability would enhance distraction of to-be-ignored speech.

An effective and well-established experimental paradigm to study the distraction of task-irrelevant, to-be-ignored speech is the *irrelevant-speech task* (e.g., Colle and Welsh, 1976; Salame and Baddeley, 1982). In brief, participants keep in working memory the serial order of stimuli while task-irrelevant sound, foremost speech, is presented. The serial recall accuracy of memory items is thought to be inversely related to the degree of distraction from task-irrelevant speech. The majority of research on the irrelevant-speech task suggests that purely acoustic factors determine the degree of distraction. As such, it has been proposed that distraction is enhanced by higher phonological similarity between memory items and task-irrelevant speech (Salame and Baddeley, 1982), or by higher variability (i.e., ‘changing state’) in the acoustic structure. The latter applies not only to task-irrelevant speech but also to task-irrelevant tones (Jones and Macken, 1993). Here, we used a variant of the irrelevant-speech task to test listeners’ distraction from a task-irrelevant speech distractor varying in spectral detail and semantic predictability.

A particularly interesting test case for the susceptibility to distraction by task-irrelevant speech is the aging listener. Older age is commonly accompanied by a decline of the functioning of the auditory system (i.e., age-related hearing loss; CHABA, 1988), but also by a general decline in cognitive capability (Park et al., 2003). Hearing loss and reduced cognitive capability, respectively, impair the perceptual segregation of concurrent sound sources and the attentional selection of target sounds, which both affect the ability to filter relevant from irrelevant auditory information (Chao and Knight, 1997; Shinn-Cunningham and Best, 2008; Passow et al., 2012). For attended speech, previous studies found that older age and hearing loss might reduce a listener’s sensitivity to temporal fine structure (Grose and Mamo, 2010; Hopkins and Moore, 2011). It might thus be expected that manipulating the degree of preserved fine structure using noise-vocoding affects speech comprehension less in older compared to younger listeners. In terms of predictability, older listeners’ speech comprehension has been found to benefit more from higher predictability, compared to younger listeners (Pichora-Fuller et al., 1995).

However, it is at present unknown how these age-differences might translate into a situation where the speech signal is not attended but ignored. It is conceivable that the more subtle manipulation of predictability of task-irrelevant speech would not be sufficient to affect the distraction in younger listeners but only in older listeners with a reduced ability to filter out irrelevant auditory information and a potentially increased susceptibility to semantic interference (Rogers et al., 2012; Obleser, 2014).

In the present study, we thus tested younger and older listeners’ memory for the serial order of spoken digits under distraction from task-irrelevant speech during memory retention. Acoustic detail (using noise-vocoding with 2, 8, or 32 frequency channels) and semantic predictability of task-irrelevant sentence endings (low or high) were varied. We asked whether more preserved acoustic detail but also higher predictability would enhance distraction by task-irrelevant speech; and whether the effects of more acoustic detail and higher predictability would be stable in the group of older listeners.

## Materials and Methods

### Participants

A sample of 22 participants, comprising 12 younger (23–33 years, 5 females) and 10 older participants (60–78 years, 7 females), took part in this study. All participants were recruited from the participant database of the Max Planck Institute for human cognitive and brain sciences in Leipzig, Germany. None of the participants reported any history of neurological diseases or significant hearing problems. Experimental procedures were approved by the local ethics committee of the University of Leipzig Medical faculty.

### Speech Materials

For the to-be-memorized stimuli, we used recordings of German spoken digits from 1 to 9 from a previous study (Obleser et al., 2012). Digits were spoken by a female voice, contained one syllable each, and had an average duration of 0.6 s. Note that the digits were acoustically intact in all experimental conditions as our acoustic manipulation (noise-vocoding) was only applied to the task-irrelevant speech materials (see below).

For the task-irrelevant speech, we used a German version of the speech in noise (SPIN) sentences (Erb et al., 2012) adopted from Kalikow et al. (1977). All sentences contained five to eight words, resulting in an average sentence duration of 2.1 s (*SD*: 0.2 s). The task-irrelevant sentences were spoken by the same female voice (fundamental frequency, *F*_0_ = 170 Hz) as the digits.

The task-irrelevant sentences varied along two orthogonal dimensions: first, sentences were spectrally degraded using 2, 8, or 32 frequency channels (2ch, 8ch, or 32ch) for noise-vocoding. In detail, 2, 8, or 32 Butterworth filters (6th order) were used to band-pass filter the speech signal. The filter centre frequencies were logarithmically spaced according to Greenwood’s cochlear frequency position function (Greenwood, 1990) and spanned the frequencies from 70 to 9000 Hz. For each frequency channel the temporal envelope was extracted using half-wave rectification and low-pass filtering (500 Hz Butterworth filter; zero-phase; 2nd order). The envelope was applied to a noise carrier that matched the cut-off frequencies of the frequency channel. For more details on the noise-vocoding procedure, see Erb et al. (2012).

For attended speech materials, a higher number of frequency channels results in enhanced intelligibility (Faulkner et al., 2001), which has also been shown for the particular sentence materials used in the present study (Hartwigsen et al., 2015). Second, the content of task-irrelevant sentences either favored one particular final word (e.g., “She covers the bed with fresh sheets”; where “sheets” is highly predictable from the sentence content) or the sentence content was not predictive of the final word (e.g., “We are very happy about the sheets”; where “sheets” is not predictable from the sentence content). For a complete list of sentences and details on the noise-vocoding procedure and the predictability manipulation, see Erb et al. (2012). All speech materials (spoken digits and noise-vocoded task-irrelevant sentences) were equalized to the same root-mean squared (rms) sound amplitude.

### Procedure

**FIGURE 1:**
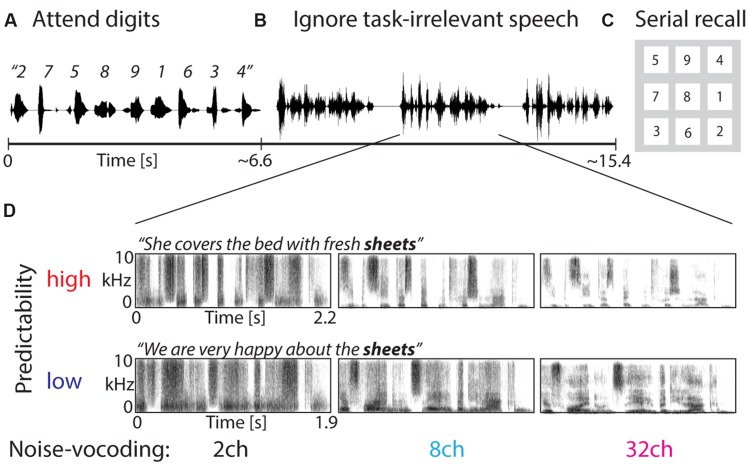
**Design of the irrelevant-speech task. (A)** Participants attended to spoken digits from 1 to 9 presented in random order. **(B)** The task was to retain the serial order of digits in memory during the presentation of task-irrelevant speech, which participants were asked to ignore. **(C)** In the end of each trial, participants had to select the digits in their order of presentation from a visually presented number pad. **(D)** Task-irrelevant speech comprised three sentences, which varied in spectral detail (using 2-channel, 8-channel, or 32-channel noise-vocoding) and predictability of sentence endings (low vs. high). The spectrograms of the task-irrelevant speech demonstrate that more spectral detail was preserved with a higher number of frequency channels used for noise-vocoding.

In the present study, we used an adapted irrelevant-speech paradigm (e.g., Colle and Welsh, 1976; Jones and Morris, 1992). On each trial, participants listened to nine spoken digits presented in random order. Spoken digits had an onset-to-onset delay of 0.75 s, resulting in an average digit presentation duration of 6.6 s, depending on the duration of the final digit (**Figure 1**). 0.5 s after the offset of the final digit, three unconnected, task-irrelevant spoken sentences were presented. On an individual trial, the three task-irrelevant sentences were equally noise-vocoded and had an equal predictability of the sentence final words. The three task-irrelevant sentences were presented with an onset-to-onset delay of 2.76 s. This resulted in a retention period of 8.28 s, during which participants retained the serial order of spoken digits in memory and ignored the task-irrelevant speech. During the presentation of speech stimuli, participants fixated a cross on the screen. After the presentation of task-irrelevant speech, participants saw a number pad with the digits from 1 to 9 on the computer screen. The ordering of digits on the number pad was randomly determined on each trial. They used the computer mouse to select the digits in the order of presentation. After the selection of an individual digit, the respective digit disappeared from the number pad. After the selection of all 9 digits from the number pad, an additional mouse click was required to start the next trial.

Prior to the experiment, participants were instructed to internally rehearse the spoken digits in their order of presentation during the presentation of task-irrelevant speech in order to keep the memory decay low. Participants were instructed to not close their eyes and to not speak the digits out loudly during a trial. Participants performed approximately 10 practice trials in order to familiarize with the task. After completion of the practice trials participants approved that they understood all instructions and were able to perform the task.

All acoustic stimuli were presented over Sennheiser HD-25 headphones. Each participant completed 120 trials, 20 for each condition in the 3 (noise-vocoding: 2ch, 8ch, 32ch) × 2 (predictability: high, low) design. Individual task-irrelevant sentences could occur more than once (at most three times) during the experiment, however, not more than once in an individual trial. The entire experiment took approximately 1 h to complete.

### Statistical Analyses

On a small proportion of trials (on average <1% of trials), participants selected an individual digit more than once during the serial recall. These trials were removed from all further analyses.

In order to assess a participant’s memory for the serial order of digits, we considered digits recalled at their respective position of presentation as “correct,” and all remaining responses as “incorrect.” The objective of this study was to test whether more acoustic (i.e., spectral) detail but also higher predictability would enhance the degree of distraction of task-irrelevant speech. Furthermore, we tested whether the degree of distraction by acoustic detail and predictability would change in a group of older (aged 60–78 years) compared with a group of younger listeners (aged 23–33 years). To this end, proportions of correctly recalled digits in the irrelevant-speech task were submitted to a repeated-measures ANOVA [within-subject factors *noise-vocoding* of task-irrelevant speech (2ch, 8ch, 32ch), *predictability* (low, high), and *digit position* (1–9); between-subject factor *age group* (younger, older)]. In case of violation of sphericity (Mauchly’s test, *p* < 0.05), we report Greenhouse-Geisser’s (GG) epsilon (ε), and GG-corrected *p*-values. As effect sizes, we report the partial eta-squared ($\eta_{p}^{2}$) for main effects and interactions in the ANOVA and *r*-equivalent (bound between 0 and 1; Rosenthal and Rubin, 2003) for *post hoc t*-tests.

Non-significant results in a null-hypothesis significance test (such as the ANOVA used here) could either stem from the absence of an effect or from the insensitivity of an analysis in detecting the effect (Dienes, 2014). To enhance the inter pretability of the non-significant effects in the present study, we calculated the Bayes Factor (BF; using R studio version 0.99.892, and the *BayesFactor* package). In detail, when comparing two statistical models, the BF indicates how many times more likely the observed data are under the alternative compared to the null-model. The BF ranges between 0 and infinity, and a BF close to 1 indicates that the data are equally likely under both models. By convention, (Jeffreys, 1939), a *BF* > 3 gives support for the alternative model, whereas a *BF* < 0.33 gives support for the null model (for a practical course on Bayesian Cognitive Modeling, see Lee and Wagenmakers, 2014).

Note that this approach also helps to overcome some of the limitations associated with the comparably small sample size available here: using a Bayes-Factor-based testing approach is recommended to specifically circumvent questions of whether the sample was simply too small to detect an effect. For instance, one recommended Bayesian procedure (see e.g., Schönbrodt et al., 2015) is to increase the sample size until a decisive BF is attained. As seen below, such decisive BFs were here clearly attained: all BFs reported here that are central to our argument were smaller than 0.33 or larger than 3.

In this study, the to-be-compared models in the Bayes-Factor comparisons comprised a selection of the following factors: random factor *participant*; fixed factors *age group*, *noise-vocoding*, *predictability*. For each Bayesian model comparison we constructed two models: first, the alternative model comprising the factor (or combination of factors) of interest and, second, the null-model lacking the factor (or combination of factors) of interest. For instance, to follow up on the non-significant main effect of predictability of task-irrelevant speech, we compared the alternative model (random factor *participant*, fixed factor *predictability*) vs. the null model with *participant* as the only factor.

## Results

**FIGURE 2:**
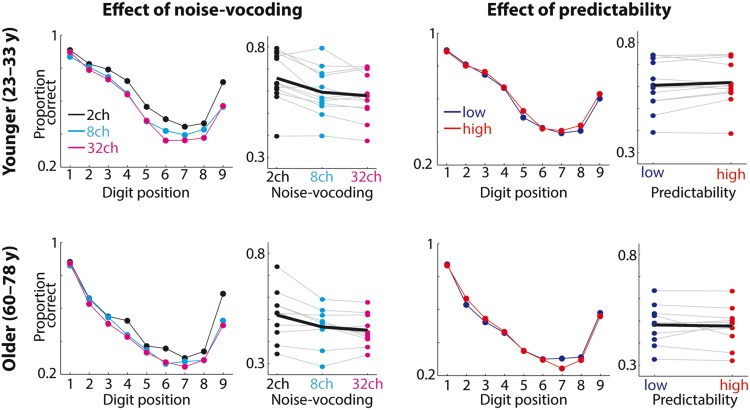
**Behavioral results.** Average proportions of correctly recalled digits for younger (top row) and older participants (bottom row) as a function of acoustic detail of task-irrelevant speech (noise-vocoding with 2, 8, or 32 channels; left) and predictability of sentence endings (low vs. high; right). Gray lines and colored dots on the right of each of the 4 subpanels show the proportions of correctly recalled digits for individual participants averaged across all (9) digit positions; the black line indicates the average across participants. The proportion of correct responses decreased with higher numbers of channels used for noise-vocoding of the task-irrelevant speech but was unaffected by the predictability of sentence endings.

**Figure 2** shows average proportions of correctly recalled digits as a function of digit position. Across experimental conditions, the proportion of correctly recalled digits was highest for digits presented at initial positions (i.e., primacy-effect) and at the final position (i.e., recency-effect), giving rise to a significant main effect of digit position (*F*_8,160_ = 66.83; GG’s ε = 0.42; *p* < 0.001; $\eta_{p}^{2}$ = 0.77).

### More Acoustic Detail of Task-Irrelevant Speech Decreases Memory Performance

**Figure 2** (left) shows that proportions of correctly recalled digits decreased with more acoustic detail of task-irrelevant speech for younger participants (mean proportion correct ± *SE*, 2ch: 0.66 ± 0.03; 8ch: 0.6 ± 0.03; 32ch: 0.58 ± 0.03) and for older participants (mean proportion correct ± *SE*, 2ch: 0.52 ± 0.04; 8ch: 0.47 ± 0.03; 32ch: 0.45 ± 0.02). The main effect of acoustic detail was highly significant (*F*_2,40_ = 25.63; *p* < 0.001; $\eta_{p}^{2}$ = 0.56; *BF* > 10^7^). Across the two age groups, mean proportions of correctly recalled digits were lower for task-irrelevant speech with 8ch compared to 2ch (*t*_21_ = 5.23; *p* < 0.001; *r* = 0.75) and for 32ch compared to 2ch (*t*_21_ = 6.41; *p* < 0.001; *r* = 0.81), but not significantly different for 32ch vs. 8ch (*t*_21_ = 1.74; *p* = 0.096; *r* = 0.36). Thus, participants were less successful in the recall of digits from memory when the to-be-ignored speech was spectrally more intact and thus more intelligible.

Furthermore, the stronger memory disruption for spectrally rich vs. degraded task-irrelevant speech was particularly evident for digits presented at later positions, underlined by a significant digit position × noise-vocoding interaction (*F*_16,320_ = 3.24; GG’s ε = 0.5; *p* = 0.002;$\eta_{p}^{2}$ = 0.14).

### Predictability of Task-Irrelevant Speech Does Not Affect Memory Performance

High- and low-predictable task-irrelevant sentence endings did not differentially affect the proportion of correctly recalled digits (**Figure 2**, right; *F*_1,20_ = 0.23; *p* = 0.635; $\eta_{p}^{2}$ = 0.01). The BF for the effect of predictability was small (*BF* = 0.2), which provides evidence against any effect of predictability of sentence endings of task-irrelevant speech on performance in the irrelevant-speech task.

Furthermore, we found no significant interaction of noise-vocoding and predictability on the proportion of correctly recalled digits (*F*_2,40_ = 0.97; *p* = 0.39; $\eta_{p}^{2}$ = 0.05; *BF* = 0.33). Thus, predictability of task-irrelevant sentences is not only ineffective in influencing task performance in and by itself, but is also unable to modulate the degree to which more acoustic detail of task-irrelevant speech decreases task performance.

### Manipulations of Task-Irrelevant Speech Similarly Affect Younger and Older Listeners

A major question in this study was whether more acoustic detail and higher predictability of task-irrelevant speech would be more performance-detrimental in older compared with younger participants. As readily visible from **Figure 2**, however, the shape of the accuracy-by-item–position graphs appears similar for the groups of younger and older participants. Accordingly, none of the interactions between age group, noise-vocoding, and predictability attained significance (age group × noise-vocoding interaction: *F*_2,40_ = 0.21; *p* = 0.811; $\eta_{p}^{2}$ = 0.01; *BF* = 0.161; age group × predictability interaction: *F*_1,20_ = 1.27; *p* = 0.272; $\eta_{p}^{2}$ = 0.06; *BF* = 0.346; age group × noise-vocoding × predictability interaction: *F*_2,40_ = 0.05; *p* = 0.947; $\eta_{p}^{2}$<0.01; *BF* = 0.21).

Irrespective of our experimental manipulations of noise-vocoding and predictability of task-irrelevant speech, the average overall performance was higher in the group of younger participants (mean proportion correct ± *SE*: 0.61 ± 0.03) than in the group of older participants (mean proportion correct ± *SE*: 0.48 ± 0.03), resulting in a significant main effect of age group (*F*_1,20_ = 9.73; *p* = 0.005; $\eta_{p}^{2}$ = 0.33; *BF* = 7.92). Furthermore, the interaction of age group and digit position was significant (*F*_8,160_ = 2.06; *p* = 0.043; $\eta_{p}^{2}$ = 0.09), driven by lower proportions of correctly recalled digits for older compared with younger listeners at digit positions 2, 3, 4, 5, and 7 (all *p* < 0.05; all *r* > 0.43) but not at remaining positions (all *p* > 0.08; all *r* < 0.38).

## Discussion

In the present study we investigated the disruption of younger and older listeners’ working memory for target speech by acoustic as well as semantic features of task-irrelevant speech. Our results can be summarized as follows: (i) Memory distraction is enhanced by higher acoustic (i.e., spectral) detail of task-irrelevant speech, but (ii) not by higher predictability of the task-irrelevant speech. (iii) Despite an overall performance decline, older listeners do not differ from younger listeners in their susceptibility to acoustic detail (i.e., high) and predictability (i.e., absent) of task-irrelevant speech.

### Memory Distraction by More Acoustic Detail of Task-Irrelevant Speech

We found that listeners’ serial recall of digits from memory was more impaired when task-irrelevant speech with more acoustic detail (with a higher number of channels used for noise-vocoding) was presented during memory retention (**Figure 2**). This is in agreement with one previous study by Ellermeier et al. (2015), which also used noise-vocoded speech in an irrelevant-speech task. Going beyond noise-vocoding, several studies using other auditory signal processing techniques have demonstrated higher distraction by acoustically more intact task-irrelevant speech (e.g., Tremblay et al., 2000; Little et al., 2010; Viswanathan et al., 2014). But can we interpret these findings with respect to listeners’ cognitive processing in the irrelevant-speech task?

Two cognitive mechanisms are involved in the irrelevant speech task. First, participants’ *attention* needs to be directed toward the serially presented items and away from the task-irrelevant speech. Second, the serial order of items needs to be maintained in *working memory* until the serial recall. Although attention and working memory are sometimes considered functionally separate cognitive mechanisms, the two at least interact strongly (Awh et al., 2006; Gazzaley and Nobre, 2012). That is, attention supports the perceptual encoding of sensory information but also promotes the post-perceptual maintenance of already encoded information in working memory (e.g., Oberauer and Hein, 2012; Lim et al., 2015). For acoustic materials such as speech and other pronounceable stimuli, the phonological loop of working memory is thought to implement their internal rehearsal (Baddeley, 1992), in order to counteract memory decay. Distraction by task-irrelevant speech thus likely occurs on the level of attention or working memory, or both. Regarding attention, to-be-ignored speech with more acoustic detail might capture attention and draw it away from items in memory. Regarding working memory, task-irrelevant speech might directly disrupt the internal serial rehearsal of digits (Jones and Morris, 1992). For both of these interpretations, task-irrelevant speech eventually taxes working memory, which is also supported by neuroimaging evidence showing that task-irrelevant speech modulates neural activity in brain areas related to working memory (Gisselgard et al., 2003, 2004).

It is important to consider that more acoustic detail enhances the intelligibility of task-irrelevant speech, which might in turn induce a stronger distraction. One previous study (Hartwigsen et al., 2015) used the same noise-vocoded sentence materials as the present study and found that speech intelligibility, quantified as the percentage of correctly recalled words, was low for 2-channel vocoded speech (<15% correct) but high for 8-channel (>90% correct) and 32-channel vocoded speech (>95% correct). In the present study we found stronger memory disruption for 8 and 32 channels compared to 2 channels, but no significant difference between the two highly intelligible 8- and 32-channel conditions. Our results thus suggest that memory disruption increases with higher intelligibility of task-irrelevant speech.

A major objective of our research is to understand the differential neural and cognitive processing of attended and ignored speech. It is well known that the processing of attended speech occupies more working memory capacity when it is acoustically degraded (e.g., noise-vocoded; Obleser et al., 2012). That is, acoustic degradation increases the listener’s need to explicitly infer linguistic content from a poor and possibly incomplete acoustic signal (e.g., Rabbitt, 1968; Rönnberg et al., 2008; Piquado et al., 2010). Interestingly, our data suggest the opposite effect if the acoustic degradation is applied to ignored speech: less degraded, and thus acoustically more detailed, ignored speech is more detrimental to the working memory processing of task-relevant materials. This observation also implies that everyday conversations should be less taxing for working memory if the attended speech signal is intact, whereas the ignored speech signal is acoustically degraded.

These different cognitive ‘fates’ of attended and ignored speech are also in line with recent neurophysiological evidence for a differential neural representation of attended and ignored speech (Ding and Simon, 2012; Mesgarani and Chang, 2012; Wöstmann et al., 2016). In essence, these studies suggest that auditory selective attention generates separable neural representations of attended and ignored speech in auditory cortex regions, which are enhanced and inhibited, respectively. Our present results are in line with this view; acoustic degradation would thus facilitate the inhibition of ignored speech.

### No Memory Distraction Resulting from Higher Predictability of Task-Irrelevant Speech

We found no evidence for an effect of predictability of task-irrelevant sentence endings on listeners’ memory for target speech (**Figure 2**, right). Importantly, a Bayesian model comparison (see Results) allows us to conclude that this result is to be interpreted as positive evidence for the lack of such an effect (as opposed to an insensitivity of our data in detecting such an effect; Dienes, 2014). We are not aware of any previous study that tested memory distraction by varying predictability of task-irrelevant sentence endings. In contrast, some studies found that semantic manipulations of task-irrelevant speech do affect the degree of distraction (Neely and LeCompte, 1999; Beaman, 2004). However, these effects were considerably smaller in size compared to acoustics manipulations.

So what can we conclude from our null-finding for predictability of task-irrelevant speech with respect to the neuro–cognitive processing of attended vs. ignored speech? Prior studies have used the identical predictability manipulation as in the current study in attended, task-relevant speech. These studies consistently found that speech comprehension increases for sentences with high vs. low predictable endings under moderate levels of noise-vocoding (Obleser et al., 2007; Hartwigsen et al., 2015; for similar evidence from bandpass-filtered speech, see Stickney and Assmann, 2001). Thus, higher predictability of attended speech does facilitate speech comprehension. Our data show that higher predictability does not affect the degree of distraction by to-be-ignored speech. This finding might suggest that the internal rehearsal of digits is not affected by predictability of task-irrelevant speech. Alternatively, a listener’s focus of attention to items in memory might be sufficiently resistant against any attentional capture from predictability of to-be-ignored speech. The latter interpretation agrees with the early-selection account of selective attention (Cherry, 1953; Broadbent, 1958; Lachter et al., 2004), which states that low-level features (such as acoustic properties) of to-be-ignored stimuli are processed and might thus affect behavior, whereas semantic features of to-be-ignored stimuli are filtered out.

It remains a possibility that our predictability manipulation was simply not potent enough (in contrast to our acoustic detail manipulation) to affect the distraction of task-irrelevant speech. In our high-predictable sentences (e.g., “She covers the bed with fresh sheets.”) only the sentence final words were predictable from the sentence context. Thus far we have tested *N* > 50 participants in the irrelevant-speech paradigm in our laboratory (for the present study and an additional unpublished study). After the experiment, participants usually report to be well aware of the acoustic but not of the predictability manipulation. We consider it possible that for a stronger semantic manipulation such as well-formed sentences (e.g., “She covers the bed with fresh sheets”) vs. semantic violations (e.g., “She covers the bed with fresh houses”) the semantic violation might draw attention away from the rehearsal of digits in memory and thus might impede memory performance.

It is important to note that the three task-irrelevant sentences on each trial in our study were unconnected. Therefore, predictability between sentences was always low whereas we manipulated solely the predictability within an individual sentence. It is thus an open question for future research whether the predictability of a single sentence or multiple connected sentences would leave distraction from task-irrelevant speech unaffected as well. Furthermore, it will be important to test the temporal dynamics of distraction from task-irrelevant speech in future studies. In the present study, the first high- vs. low-predictable sentence final word occurred more than approximately 1.5 s after the onset of the task-irrelevant speech. This is relatively late compared to the manipulation of acoustic detail, which affected the task-irrelevant speech from beginning on. We can thus not exclude that the present predictability manipulation occurred too late during memory retention to disrupt already consolidated memory content.

### Older Age Does Not Promote Distraction by Acoustics and Predictability of Ignored Speech

Overall performance in the irrelevant-speech task was poorer for older compared with younger listeners, which is in agreement with the general trajectory of decline in sensory and memory functions at older age (e.g., Fisk and Warr, 1996; Baltes and Lindenberger, 1997). More importantly, we found that the pattern of results – enhanced distraction by more acoustic detail but not by higher predictability of task-irrelevant speech – was indistinguishable in the groups of older (60–78 years) and younger listeners (23–33 years; **Figure 2**). This is in line with previous studies that found no age-differences regarding the degree of distraction in the irrelevant-speech task (e.g., Rouleau and Belleville, 1996; Bell and Buchner, 2007). There is, however, some evidence that meaningful vs. non-meaningful to-be-ignored speech might distract older listeners’ more than younger listeners (Tun et al., 2002). Note, however, that the present semantic manipulation of predictability of sentence endings is arguably more subtle and thus more ecologically valid compared to the operationalization of meaningless speech as randomly ordered word lists or speech in an unfamiliar language, as used by Tun et al. (2002). This difference might in parts explain the absence of an age effect for distraction by predictability of task-irrelevant speech in the present study.

In general, the ability to focus attention on target stimuli and to ignore irrelevant information (i.e., *attentional control*) decreases at older age (e.g., Shinn-Cunningham and Best, 2008; Passow et al., 2012). Does the absence of an age effect in the present study thus suggest that our older listeners did not show any substantial decline of attentional control? We deem it more likely that a decline in attentional control is not necessarily a limiting factor in the irrelevant-speech task. This favors the view that task-irrelevant speech directly impairs working memory, without this effect being mediated by the attentional capture of task-irrelevant speech. In sum, our main conclusion holds across the two age groups tested in this study: not all features that make attended speech easier to comprehend necessarily enhance distraction if speech is being ignored. There is stronger distraction by task-irrelevant speech with more acoustic detail, but not with higher predictability.

## Conclusion

The presentation of task-irrelevant speech can disrupt memory for relevant information. We show here that the features that make attended speech easier to process are not the same that render ignored speech more distracting. That is, only a higher degree of acoustic detail but not higher predictability enhance the degree of distraction by to-be-ignored speech. This finding is preserved even if the attentional control ability decreases in healthy aging.

## Author Contributions

MW and JO designed research; MW performed research; MW analyzed data; MW and JO wrote the paper.

## Conflict of Interest Statement

The authors declare that the research was conducted in the absence of any commercial or financial relationships that could be construed as a potential conflict of interest.
